# Nationwide variability in sedation, analgesia, and developmental care practices during neonatal therapeutic hypothermia: A French national survey

**DOI:** 10.1007/s00431-026-07150-8

**Published:** 2026-06-11

**Authors:** Claire Osswald, Kévin Le Duc, Laurence Chaton, Sylvie Joriot, Laurent Storme, Florence Flamein, Mohamed Riadh Boukhris

**Affiliations:** 1 CHU Lille, Department of Neonatology, Jeanne de Flandre Hospital, F-59000 Lille, France; 2 Univ. Lille, CHU Lille, ULR 2694 METRICS: Evaluation Des Technologies de Santé Et Des Pratiques Médicales - Perinatal Environment and Health, F-59000 Lille, France; 3 CHU Lille, Department of Clinical Neurophysiology, Roger Salengro Hospital, F-59000 Lille, France; 4 CHU Lille, Department of Pediatric Neurology, Roger Salengro Hospital, F-59000 Lille, France; 5 CHU Lille, Clinical Research and Investigation Center, Jeanne de Flandre Hospital, F-59000 Lille, France

**Keywords:** Therapeutic hypothermia, Hypoxic–ischemic encephalopathy, Sedation, Analgesia, Neonatal intensive care units, Survey

## Abstract

Analgesia, sedation and developmental care are key components of supportive management for newborns with hypoxic–ischemic encephalopathy (HIE) undergoing therapeutic hypothermia (TH). However, evidence-based recommendations remain limited, and practices may vary substantially between centers. We aimed to describe current practices in analgesia, sedation, and developmental care during TH across French neonatal intensive care units (NICUs). We conducted a nationwide cross-sectional survey using a standardized electronic questionnaire distributed to all NICUs performing TH in France. Each center provided a single consolidated response. Descriptive statistics were used. Fifty-one of 63 eligible NICUs responded (80%), including 33 university and 18 general hospitals. Formal developmental care programs were available in 88% of centers. Routine endotracheal intubation during TH was reported by 71%. The most frequently used medications were midazolam (94%), sufentanil (88%), morphine (84%), and paracetamol (82%). Analgesia–sedation regimens and dosing strategies varied markedly across centers. Most NICUs (75%) expressed interest in developing protocols allowing TH without routine intubation.

*Conclusions*: Substantial inter-center variability exists in analgesia, sedation, and developmental care practices during neonatal TH. These findings support the need for standardized, evidence-based protocols to optimize neonatal comfort and reduce practice heterogeneity.

**Table:** 

**What is Known:** • *Therapeutic hypothermia is the standard neuroprotective treatment for moderate-to-severe neonatal hypoxic-ischemic encephalopathy*. •* Analgesia, sedation, and supportive care during therapeutic hypothermia are widely used, but evidence-based recommendations remain limited*.	
**What is New:** • *This first nationwide French survey demonstrates marked inter-center variability in analgesia, sedation, developmental care, and routine intubation practices during neonatal therapeutic hypothermia*. • *These findings highlight the need for standardized, pharmacologically informed protocols during therapeutic hypothermia, prioritizing medications with more predictable metabolism and clearer pharmacokinetic-based dosing strategies*.

**Supplementary Information:**

The online version contains supplementary material available at 10.1007/s00431-026-07150-8.

## Introduction

Therapeutic hypothermia (TH) is currently the only evidence-based neuroprotective therapy recommended for neonates with moderate-to-severe hypoxic–ischemic encephalopathy (HIE) [1]. When initiated within the first 6 h of life and maintained for 72 h, TH reduces mortality and neurodevelopmental impairment. However, outcomes remain suboptimal, with reported mortality rates of 15–25% and long-term neurodevelopmental disabilities affecting 25–30% of survivors, including cerebral palsy, cognitive impairment, and learning difficulties [2, 3].

Supportive care is a key component of the management of infants undergoing TH. Neonates admitted to intensive care are exposed to an average of approximately 15 painful procedures per day [4]. Infants undergoing TH may experience additional sources of discomfort, including cold exposure, repeated blood sampling, intubation, mechanical ventilation and other intensive care interventions [5]. Repeated exposure to painful stimuli during the neonatal period has been associated with altered pain processing, impaired brain maturation, and adverse neurodevelopmental outcomes [6, 7].

Supportive care during TH includes both pharmacological interventions (analgesia and sedation) and non-pharmacological approaches aimed at reducing stress and discomfort. These measures may include developmental care, environmental modulation, positioning, and minimal handling. Appropriate assessment of pain and discomfort is therefore essential. Numerous neonatal pain assessment tools have been developed, including behavioral scales and multidimensional instruments combining behavioral and physiological parameters, although none has been specifically validated for infants undergoing TH [5].

Despite the widespread implementation of TH, no standardized recommendations specifically address analgesia, sedation, and developmental care during hypothermia, and practices may vary substantially between centers. In France, the most recent recommendations specifically addressing TH were published more than a decade ago [8].

The objective of this study was therefore to describe national practices and assess variability in analgesia, sedation, and developmental care, including both pharmacological and non-pharmacological interventions**,** during TH in neonates with HIE across French neonatal intensive care units.

## Methods

### Study design and survey development

We conducted a nationwide cross-sectional survey to describe analgesia, sedation, pain assessment, and developmental care practices during TH in neonates with HIE. The survey focused on supportive care practices during TH and did not specifically assess anticonvulsant treatment strategies or dosing regimens.

The questionnaire was developed following a literature review to identify clinically relevant domains and key practice indicators. The initial version was reviewed by clinicians from the HIE reference group at Lille University Hospital and subsequently pre-tested by neonatal intensive care unit (NICU) clinicians at Lille and Rouen University Hospitals who were not involved in its development. The questionnaire was revised according to their feedback. Content validity was further evaluated by neonatologists and NIDCAP-certified nurses, who assessed the relevance, comprehensiveness, and clarity of the survey items.

The study was designed and reported in accordance with the STROBE (Strengthening the Reporting of Observational Studies in Epidemiology) guidelines for cross-sectional studies and the CHERRIES (Checklist for Reporting Results of Internet E-Surveys) checklist for internet-based surveys.

### Survey content

The final version comprised 54 questions covering demographic characteristics, NICU organization, TH practices, environmental factors, and analgesia–sedation management during hypothermia. Question formats included single-choice, multiple-choice, and short-answer items (Supplementary Material).

Key terms used in the questionnaire were defined as follows. The Newborn Infant Parasympathetic Evaluation (NIPE) monitor is a non-invasive tool that analyzes heart rate variability to estimate parasympathetic activity, providing a continuous index reflecting autonomic balance and comfort in neonates. The Newborn Individualized Developmental Care and Assessment Program (NIDCAP) is an individualized developmental care approach based on detailed observation of infant behavior to guide caregiving and environmental adaptations. The Family and Infant Neurodevelopmental Education (FINE) program is a structured educational framework aimed at promoting developmental and family-centered care practices in neonatal settings.

### Setting and participants

The survey was distributed to all 63 NICUs performing TH among the 67 NICUs across mainland France and overseas territories. Each unit designated a referent clinician to complete the questionnaire. Eligible respondents included hospital practitioners, academic physicians, and attending neonatologists.

### Survey administration and data collection

The questionnaire was distributed electronically via Google Forms® (Google LLC, 2023) between December 20, 2022, and March 21, 2023. Invitations were sent directly to the designated senior clinician of each eligible NICU. Reminder emails and follow-up telephone contacts were conducted to maximize the response rate. Completion time was estimated at approximately 10–15 min. Mandatory fields were used for key items, whereas optional items could be skipped.

Duplicate entries were screened manually using center identity, respondent characteristics, and timestamps. When multiple responses were received from the same center, concordance was assessed and a single consolidated response was retained.

No questionnaires were excluded because of missing data. Responses were reviewed for completeness before analysis.

### Statistical analysis

Data were summarized using descriptive statistics. Categorical variables are presented as counts and percentages. For maintenance infusion doses, medians and interquartile ranges were calculated and visualized using boxplots displayed on a logarithmic scale because of the wide range of reported values. Given the descriptive nature of this nationwide survey, no prespecified inferential statistical analyses were planned. Exploratory post hoc comparisons were performed using Fisher’s exact test for selected variables when clinically relevant. Statistical significance was defined as a two-sided p value < 0.05.

## Results

### Responding centers

Of the 67 NICUs in France, 63 provided TH and were eligible for participation. Fifty-one centers responded (81%), including 33 university hospitals (65%) and 18 general hospitals (35%). Characteristics of participating centers and respondents are summarized in Table 1.

**Table 1:** Characteristics of participating centers and physicians (*n* = 51)

Variable	*n *(%)
Center characteristics	
Neonatal and pediatric intensive care	13 (25.5)
Neonatal intensive care only	38 (74.5)
Activity level – Births per year	
1000–2500	10 (19.6)
2500–4000	28 (54.9)
> 4000	13 (25.5)
Therapeutic hypothermia cases per year	
< 5	5 (9.8)
5–10	12 (23.5)
10–15	19 (37.3)
> 15	15 (29.4)
Organization	
Dedicated HIE referent clinician	24 (47.1)
Updated local HIE guidelines	44 (86.2)
Physician experience – Neonatology	
< 5 years	2 (4.0)
5–10 years	14 (27.4)
> 10 years	21 (41.2)
Physician experience with therapeutic hypothermia	
< 5 years	2 (4.0)
5–10 years	19 (37.2)
> 10 years	30 (58.8)

### Therapeutic hypothermia and environment

Environmental adaptations during TH varied according to local resources (Table 2). Among the 45 NICUs with a formal developmental care policy, 48.9% implemented NIDCAP, 24.5% the Bullinger approach, 2.2% both approaches, 13.3% FINE program, and 11.1% basic developmental care practices.

**Table 2:** Environmental and developmental care during therapeutic hypothermia (*n* = 51)

Variable	*n *(%)
Single-room organization available	48 (94.1)
Mother–infant room available	16 (31.4)
24-h parental presence allowed	50 (98.0)
Developmental care implemented	45 (88.2)
Noise reduction strategies	45 (88.2)
Light reduction strategies	47 (92.2)
Four-handed care routinely/frequently performed	36 (70.6)
Parent involved in care during procedures	23 (45.1)
Holding infant during TH allowed	10 (19.6)

Measures to reduce sensory stimulation were commonly reported. Noise reduction strategies included single-family rooms (84.4%), reduced alarm volumes (71.1%), and earplugs (35.6%). Light exposure was limited through lowered blinds (83%), adjustable lighting (93.6%), and phototherapy eye protection (34%).

### Therapeutic hypothermia and intubation

**Fig. 1:**
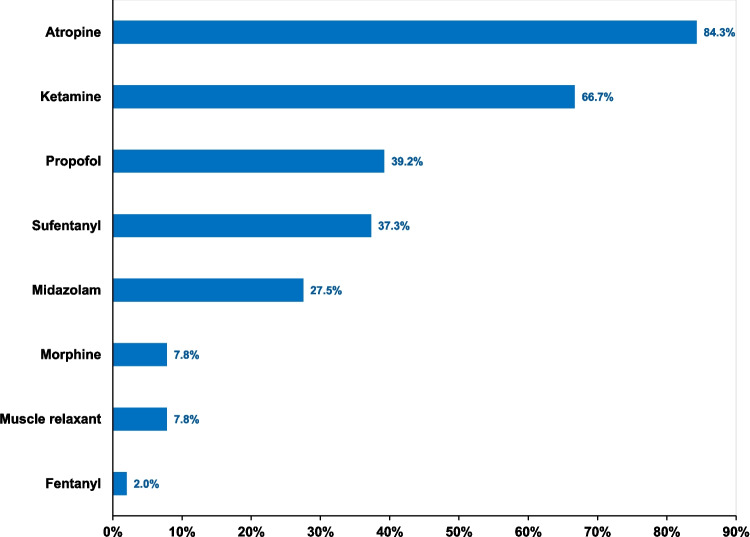
Frequency of medications administered for tracheal intubation in neonatal intensive care units caring for neonates undergoing therapeutic hypothermia

Routine endotracheal intubation during TH was reported by 70.6% of centers. Indications included optimization of neuroprotection (75.0%), management of drug-induced apnea (72.2%), adherence to local NICU protocols (16.7%), and patient comfort (5.6%). Ketamine combined with atropine was the most frequently reported intubation regimen (Fig. 1). Figure 1 refers exclusively to medications administered during tracheal intubation and does not include maintenance analgesia or sedation regimens during TH.

Overall, 74.5% of NICUs expressed interest in developing protocols to perform TH without routine intubation; however, 63.2% of these centers currently practiced systematic intubation.

### Pain assessment

Seventy-two percent of NICUs reported local pain management guidelines, of which 49.0% had been updated within the past five years. For infants with HIE undergoing TH, 60.8% of centers had dedicated guidelines, including 37.3% updated within five years.

Pain was primarily assessed using the COMFORT-B and EDIN (Échelle de Douleur et d’Inconfort du Nouveau-né) scales. In most centers, assessments were performed every 3 h and adjusted according to clinical status. Continuous monitoring tools were used in 21.6% of NICUs, including the Newborn Infant Parasympathetic Evaluation (NIPE) (15.7%), skin conductance (2.0%), both modalities (2.0%), or near-infrared spectroscopy (2.0%).

### Analgesic and sedative drugs

**Fig. 2:**
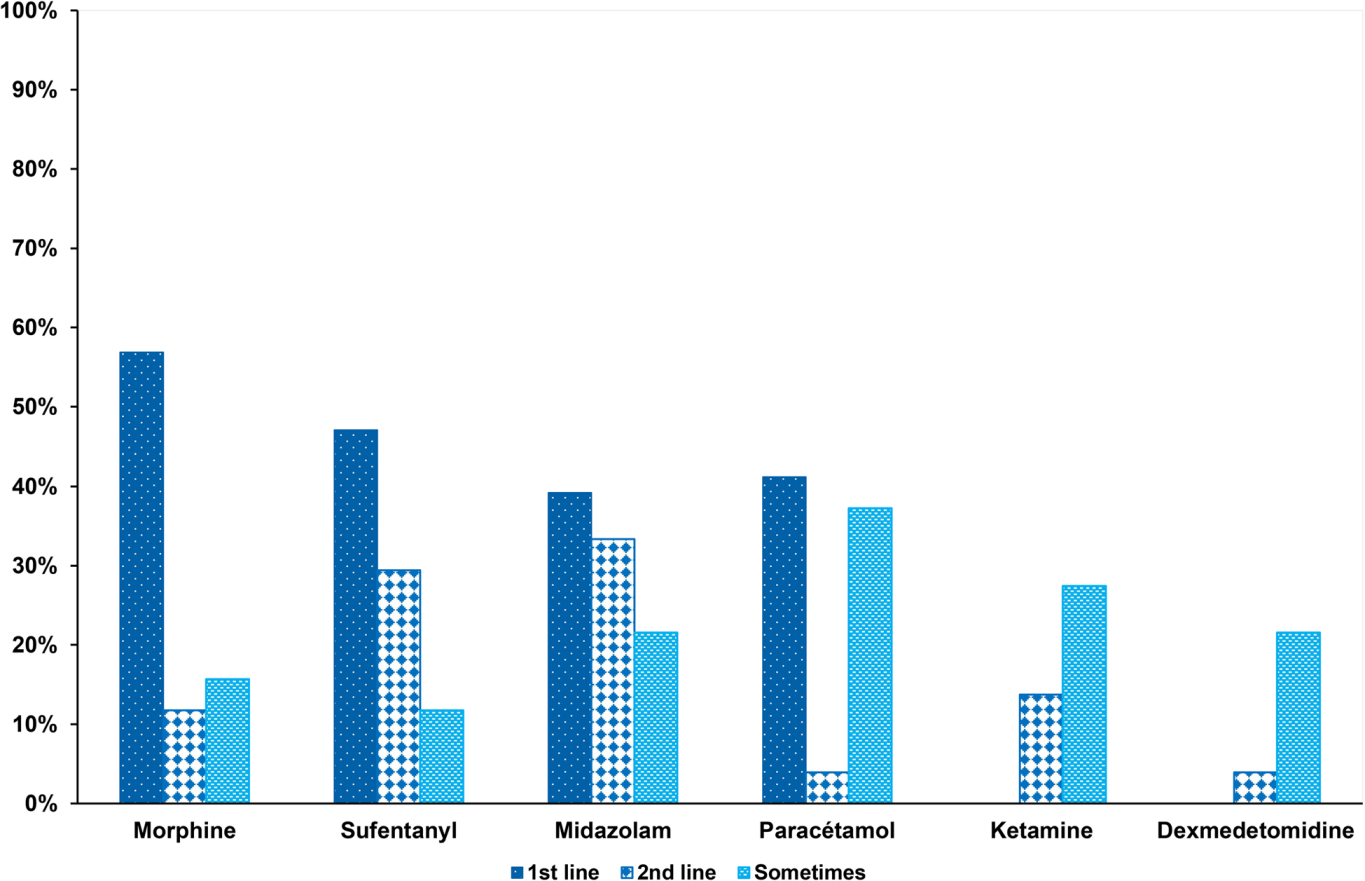
Frequency of analgesic and sedative medications administered during therapeutic hypothermia across neonatal intensive care units

Analgesic or sedative therapy was primarily initiated to improve comfort and support neuroprotection. The most frequently used agents were idazolam (94%), sufentanil (88%), morphine (84%), and paracetamol (82%). (Fig. 2). Dexmedetomidine was used in 25.5% of centers, mainly as a second-line or occasional treatment, whereas nefopam, remifentanil, fentanyl, propofol, oral glucose, and neuromuscular blocking agents were rarely used or not used. Dexmedetomidine use did not differ significantly between centers with and without routine intubation practices (8/36 vs. 5/15; Fisher’s exact test, p = 0.63).

At TH initiation, medications were administered as continuous infusions in 51.0% of NICUs and as continuous infusions with supplemental boluses in 47.0%. By 24 h, continuous infusion alone was the most common strategy (66.7%), whereas one center reported using boluses only. Transition to bolus-only administration remained uncommon and was reported by 7.8% of NICUs after 72 h.

Dose adjustments were mainly guided by pain scale scores (96.1%), caregiver assessment (70.6%), and clinical examination (43.1%), whereas pharmacokinetic considerations were reported by only 9.8% of centers. At 24 h, doses were increased in 31.4% of centers, unchanged in 47.1%, and decreased in 21.6%.

**Fig. 3:**
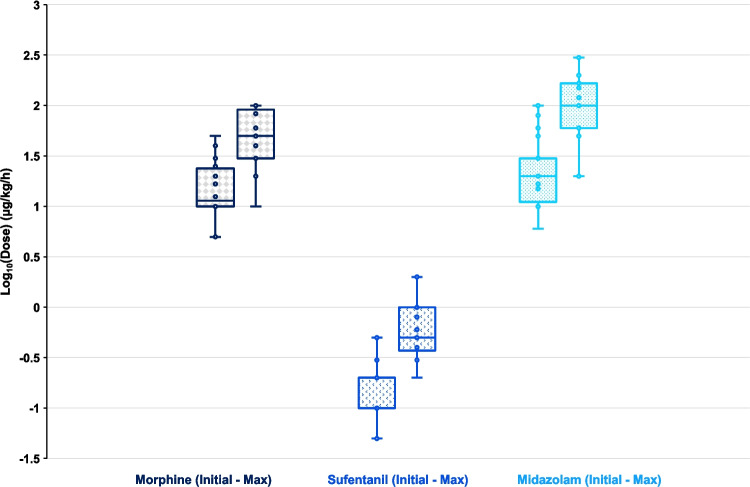
Variability in maintenance infusion doses of commonly used analgesic and sedative agents during therapeutic hypothermia. Boxplots showing the distribution of initial maintenance doses and maximum maintenance doses reported by participating neonatal intensive care units (NICUs) for morphine, sufentanil, and midazolam during therapeutic hypothermia. Doses are expressed in µg/kg/h and displayed on a logarithmic (log10) scale. The central line represents the median, boxes indicate the interquartile range, whiskers represent the range of non-outlier values, and individual points correspond to participating NICUs. Reported doses refer exclusively to maintenance infusion regimens used for analgesia and sedation during therapeutic hypothermia. Data on intermittent bolus doses, medications administered for tracheal intubation, and anticonvulsant treatment regimens are not represented in this figure

For continuous maintenance infusion during TH, morphine, sufentanil, and midazolam were used by 62.7%, 64.7%, and 54.9% of participating NICUs, respectively. Marked inter-center variability was observed in both initial and maximum maintenance infusion doses for each agent, as illustrated in Fig. 3. The complete range of reported initial and maximum doses for all surveyed medications is provided in Table S1.

Medication changes were primarily attributed to insufficient efficacy (92.2%) or adverse effects (68.6%). EEG-related concerns, including suppression of background activity on continuous EEG or amplitude-integrated EEG, were less frequently reported (15.7% and 5.9%, respectively).

Treatment discontinuation most commonly occurred at extubation (47.1%), at the end of rewarming (31.4%), or upon completion of TH (15.7%). Monitoring for withdrawal syndrome after treatment discontinuation was inconsistent, being reported as systematic in 2.0% of NICUs, frequent in 3.9%, occasional in 39.2%, rare in 39.2%, and absent in 3.9%.

## Discussion

This nationwide survey provides a comprehensive overview of analgesia, sedation, pain assessment, and developmental care practices during TH for neonates with HIE in France. Despite the widespread implementation of local protocols, we observed marked inter-center variability in developmental care strategies, pain assessment methods, respiratory management, and analgesic–sedative regimens. Similar heterogeneity has been reported internationally and likely reflects the absence of universally accepted evidence-based recommendations regarding supportive care during TH [5, 9].

Altered drug disposition during TH likely contributes to the variability in sedation practices observed across centers. Hypothermia may affect hepatic enzymatic activity, renal elimination, protein binding, and organ perfusion, thereby increasing the risk of drug accumulation and prolonged exposure [10]. These effects are further complicated by developmental immaturity of neonatal hepatic and renal clearance pathways [11]. Morphine clearance is reduced during TH, and doses exceeding 10 µg/kg/h between 24 and 72 h of cooling may result in potentially toxic plasma concentrations [12]. In our survey, morphine, sufentanil, and midazolam were the most frequently used agents, with considerable variability in both initial and maximum maintenance infusion doses. Midazolam exposure may also vary because of developmental CYP3A immaturity and accumulation of pharmacologically active metabolites. In addition, co-administration of phenobarbital may alter midazolam pharmacokinetics through CYP3A induction [13].

The variability observed in maintenance infusion doses may reflect differing local approaches to dose adaptation during TH. Similar heterogeneity has recently been reported in a single-center retrospective study of neonates undergoing TH, which highlighted substantial variability in sedative and analgesic exposure and suggested that treatment reassessment may not always adequately account for evolving pharmacokinetic changes during recovery [14]. However, because our survey did not specifically investigate intentional dose-reduction strategies, these observations should be interpreted cautiously. Future standardized protocols should incorporate pharmacokinetic considerations, individualized dose titration, and multimodal pain assessment strategies. Based on current evidence, future guidelines should integrate multimodal pain assessment, pharmacokinetically informed opioid titration, cautious use of benzodiazepines as adjunctive agents, and individualized respiratory management. Based on current pharmacokinetic and safety data, opioid-based regimens may represent the most commonly supported first-line pharmacological approach during TH, whereas benzodiazepines should be used cautiously and mainly as adjunctive agents because of concerns regarding altered pharmacokinetics, hemodynamic instability, and interference with neurological assessment. Further studies are needed to establish evidence-based dosing recommendations and validate pain and sedation assessment tools specifically for infants undergoing TH [12, 13].

Dexmedetomidine, an α2-adrenergic agonist, is emerging as a potential alternative for neonatal sedation and analgesia. In our study, 25.5% of centers reported occasional or second-line dexmedetomidine use. Available observational studies suggest that dexmedetomidine is feasible and generally well tolerated in neonates undergoing TH, with bradycardia being the most frequently reported adverse effect and limited respiratory compromise [15–17]. In addition, dexmedetomidine may reduce exposure to opioids and benzodiazepines [15–17]. However, its neuroprotective profile remains uncertain. Experimental studies have reported potentially neuroprotective effects [18], although the effects of dexmedetomidine on the developing brain remain incompletely understood. Furthermore, dexmedetomidine may be associated with hemodynamic adverse effects, including bradycardia and hypotension [20]. Therefore, although promising, its safety, optimal dosing strategies, and long-term neurodevelopmental effects require further investigation before widespread adoption can be recommended.

Non-pharmacological interventions are increasingly recognized as important components of supportive care during TH. Developmental care, reduction of environmental stimuli, positioning, facilitated tucking, non-nutritive sucking, and parental involvement may improve comfort and reduce stress during cooling. In our survey, 19.6% of centers reported allowing skin-to-skin or “in-arms” positioning during TH. Although evidence remains limited, these approaches appear feasible in clinically stable infants and may strengthen parent–infant bonding [21, 22].

Pain assessment during TH remains particularly challenging. The EDIN and COMFORT-B scales were the most commonly used tools in our survey, yet none of the currently available neonatal pain and sedation assessment tools has been specifically validated in neonates undergoing TH. Interpretation may be confounded by sedation, altered neurological status, mechanical ventilation, and the physiological effects of hypothermia itself. These limitations support the use of a multimodal approach to pain and discomfort assessment during TH, integrating behavioral scales, physiological parameters, neurological evaluation, and contextual clinical assessment rather than relying on a single tool alone, as previously proposed in other neonatal populations [23]. The use of non-validated tools may contribute to both under-treatment of discomfort and oversedation. Oversedation represents a potential iatrogenic risk because it may contribute to hypotension, prolonged ventilation, impaired neurological examination, and altered EEG interpretation [10, 13, 24]. Hoffman et al. reported increased stress responses despite low behavioral scores, suggesting that conventional pain scales may underestimate discomfort during TH [25]. Conversely, implementation studies have suggested that the N-PASS scale may be useful for pain and sedation assessment in this setting [26]. Further studies are needed to validate pain and sedation assessment tools specifically in infants undergoing TH.

Interestingly, nearly one-third of NICUs in our survey did not routinely intubate infants during TH. This finding likely reflects the absence of specific French or international recommendations regarding routine tracheal intubation practices and standardized first-line sedation strategies during cooling. Routine intubation is not without potential risks. In infants without primary respiratory disease, mechanical ventilation may induce hypocapnia, which has been associated with adverse neurological outcomes in neonatal HIE [27]. Moreover, ventilation itself may increase stress, prolong sedative exposure, and necessitate higher drug dosages, potentially complicating neurological assessment and EEG interpretation. These findings support further evaluation of non-intubated TH strategies in appropriately selected infants.

This study provides important insights into current supportive care practices during TH in France. A major strength is the high participation rate, with responses obtained from 81% of eligible NICUs, providing near-exhaustive national coverage. Nevertheless, several limitations should be acknowledged. First, survey-based studies are subject to reporting bias and may not fully reflect actual bedside practices. Second, our survey focused primarily on supportive care and did not specifically evaluate seizure management or hemodynamic strategies, both of which may influence sedation requirements. Third, although some practices may be influenced by country-specific organizational structures and local protocols, similar heterogeneity in sedation, analgesia, and pain assessment practices has been reported in multinational cohorts such as EUROPAIN and in recent reviews of TH practices [5, 9]. These observations suggest that the challenges identified in our survey are not specific to the French healthcare system. Therefore, although absolute frequencies and specific therapeutic preferences may vary across countries, the broader relevance and transferability of our findings are likely to extend beyond the French setting.

Future studies should better define pharmacokinetic-based dosing strategies, validate multimodal pain assessment approaches during TH, and evaluate the long-term neurodevelopmental impact of different sedation regimens. Expert consensus processes, such as Delphi-based initiatives, may help establish standardized evidence-based recommendations integrating pharmacological, developmental, and pain-assessment strategies while minimizing unnecessary sedative exposure.

Pain and discomfort management remain key challenges during TH. Practice heterogeneity identified in this national survey highlights the need for harmonized, evidence-based supportive care recommendations to optimize both neonatal comfort and neurological outcomes.

## Supplementary Information

Below is the link to the electronic supplementary material.

Supplementary file1 (DOCX 15 KB)

## Data Availability

The dataset generated and/or analyzed during the current study are available from the corresponding author on reasonable request.
