# Supplementary material for: Nationwide variability in sedation, analgesia, and developmental care practices during neonatal therapeutic hypothermia: A French national survey
Source: Eur J Pediatr. 2026 Jun 11;185(7):487. doi: 10.1007/s00431-026-07150-8 (PMC13260189; doi:10.1007/s00431-026-07150-8)
Supplement: Supplementary file 1 — Supplementary file1 (DOCX 15 KB) [file 431_2026_7150_MOESM1_ESM.docx]

**Table S1.** Reported ranges of maintenance infusion doses for analgesic and sedative medications during therapeutic hypothermia

| **Drug** | **Initial maintenance dose** | **Maximum maintenance dose** | **Unit** |
| --- | --- | --- | --- |
| **Morphine** | 5–50 | 20–100 | µg/kg/h |
| **Sufentanil** | 0.05–0.5 | 0.2–2 | µg/kg/h |
| **Midazolam** | 6–100 | 20–300 | µg/kg/h |
| **Ketamine** | 0.1–0.5 | 0.5–2 | mg/kg/h |
| **Dexmedetomidine** | 0.2–0.5 | – | µg/kg/h |
| **Fentanyl** | 0.2–5 | 1–20 | µg/kg/h |
| **Atracurium** | 0.5 | – | mg/kg/h |
| **Cisatracurium** | 0.1–0.2 | – | mg/kg/h |
